# Supplementary material for: Impact of surgical approaches on long-term survival outcomes of patients with pancreatic neuroendocrine carcinoma
Source: PLoS One. 2025 Mar 24;20(3):e0319906. doi: 10.1371/journal.pone.0319906 (PMC11932467; doi:10.1371/journal.pone.0319906)
Supplement: Supplementary Table 1 — (DOCX) [file pone.0319906.s001.docx]

**Supplementary Table 1.** Demographic and clinical characteristics of patients before multiple imputation

| Variables | Total  (n=1,331) | LR group  (n=678) | RR group  (n=653) | *P*-value |
| --- | --- | --- | --- | --- |
| **Sex, n (%)** |  |  |  | 0.341 |
| Male | 731 (54.9%) | 381 (56.2%) | 350 (53.6%) |  |
| Female | 600 (45.1%) | 297 (43.8%) | 303 (46.4%) |  |
| **Age, years, n (%)** |  |  |  | 0.688 |
| <60 | 623 (46.8%) | 321 (47.3%) | 302 (46.2%) |  |
| ≥60 | 708 (53.2%) | 357 (52.7%) | 351 (53.8%) |  |
| **Race, n (%)** |  |  |  | 0.999 |
| White | 1,046 (78.6%) | 533 (78.6%) | 513 (78.6%) |  |
| Black | 140 (10.5%) | 71 (10.5%) | 69 (10.6%) |  |
| Others | 138 (10.4%) | 70 (10.3%) | 68 (10.4%) |  |
| Unknown | 7 (0.5%) | 4 (0.6%) | 3 (0.5%) |  |
| **Grade, n (%)** |  |  |  | **<0.001** |
| Well | 921 (69.2%) | 494 (72.9%) | 427 (65.4%) |  |
| Moderately | 217 (16.3%) | 109 (16.1%) | 108 (16.5%) |  |
| Poorly | 71 (5.3%) | 15 (2.2%) | 56 (8.6%) |  |
| Undifferentiated | 14 (1.1%) | 3 (0.4%) | 11 (1.7%) |  |
| Unknown | 108 (8.1%) | 57 (8.4%) | 51 (7.8%) |  |
| **Marital status, n (%)** |  |  |  | 0.849 |
| Married | 850 (63.9%) | 438 (64.6%) | 412 (63.1%) |  |
| Unmarried | 411 (30.9%) | 205 (30.2%) | 206 (31.5%) |  |
| Unknown | 70 (5.3%) | 35 (5.2%) | 35 (5.4%) |  |
| **Tumor size, cm, n (%)** |  |  |  | **<0.001** |
| ≤2.0 | 523 (39.3%) | 302 (44.5%) | 221 (33.8%) |  |
| 2.1-5.0 | 574 (43.1%) | 276 (40.7%) | 298 (45.6%) |  |
| >5.0 | 210 (15.8%) | 85 (12.5%) | 125 (19.1%) |  |
| Unknown | 24 (1.8%) | 15 (2.2%) | 9 (1.4%) |  |
| **AJCC stage, n (%)** |  |  |  | **<0.001** |
| I | 797 (59.9%) | 470 (69.3%) | 327 (50.1%) |  |
| II | 475 (35.7%) | 184 (27.1%) | 291 (44.6%) |  |
| III | 29 (2.2%) | 4 (0.6%) | 25 (3.8%) |  |
| Unknown | 30 (2.3%) | 20 (2.9%) | 10 (1.5%) |  |
| **T stage, n (%)** |  |  |  | **<0.001** |
| T1 | 479 (36.0%) | 285 (42.0%) | 194 (29.7%) |  |
| T2 | 452 (34.0%) | 242 (35.7%) | 210 (32.2%) |  |
| T3 | 354 (26.6%) | 132 (19.5%) | 222 (34.0%) |  |
| T4 | 22 (1.7%) | 3 (0.4%) | 19 (2.9%) |  |
| TX | 24 (1.8%) | 16 (2.4%) | 8 (1.2%) |  |
| **N stage, n (%)** |  |  |  | **<0.001** |
| N0 | 994 (74.7%) | 558 (82.3%) | 436 (66.8%) |  |
| N1 | 320 (24.0%) | 110 (16.2%) | 210 (32.2%) |  |
| NX | 17 (1.3%) | 10 (1.5%) | 7 (1.1%) |  |
| **Radiation, n (%)** |  |  |  | **0.005** |
| None/Unknown | 1,300 (97.7%) | 670 (98.8%) | 630 (96.5%) |  |
| Yes | 31 (2.3%) | 8 (1.2%) | 23 (3.5%) |  |
| **Chemotherapy, n (%)** |  |  |  | **<0.001** |
| No/Unknown | 1,248 (93.8%) | 657 (96.9%) | 591 (90.5%) |  |
| Yes | 83 (6.2%) | 21 (3.1%) | 62 (9.5%) |  |
| **Survival months, median (IQR)** | 77 (34, 102) | 76 (40, 101) | 78 (30, 102) | 0.315 |

LR: local resection; RR: radical resection; Others: American Indian, Alaska Native, Asian/Pacifc Islander; IQR: interquartile range; bold values indicate *P* < 0.05
