# Supplementary material for: Impact of surgical approaches on long-term survival outcomes of patients with pancreatic neuroendocrine carcinoma
Source: PLoS One. 2025 Mar 24;20(3):e0319906. doi: 10.1371/journal.pone.0319906 (PMC11932467; doi:10.1371/journal.pone.0319906)
Supplement: Attachment — Submitted filename: Point by Point Response R1.docx [file pone.0319906.s002.docx]

Dear editor and reviewers of PLOS ONE:

Title: Impact of Surgical Approaches on Long-term Survival Outcomes of Patients with Pancreatic Neuroendocrine Carcinoma

By: zhenguo qiao et al

Thank you very much for your letter and for the editors’ and reviewers’ comments concerning our manuscript entitled “Impact of Surgical Approaches on Long-term Survival Outcomes of Patients with Pancreatic Neuroendocrine Carcinoma”. These comments are of great reference value to the revision and improvement of our paper and have important guiding significance to our researches. We have studied comments carefully and have made correction. We hope that the revision is acceptable and look forward to hearing from you soon. Revised portion are marked in color in the paper. The main corrections in the paper and the responds to the reviewer’s comments are as flowing:

Review Comments to the Author

Please use the space provided to explain your answers to the questions above. You may also include additional comments for the author, including concerns about dual publication, research ethics, or publication ethics. (Please upload your review as an attachment if it exceeds 20,000 characters)

Reviewer #1: This paper is of great significance in discussing the influence of surgical methods on the survival outcome of PNEC patients, but there is still room for improvement in research design, data analysis and discussion of results.

**Comment 1**

Although Cox proportional hazard model is used for multivariate analysis, the reasons for model selection and the results of hypothesis testing are lack of detailed explanation. It is suggested that the author provide more details about statistical analysis in the method part, including how to deal with missing data and the criteria for choosing variables.

**Response 1**

Thank you for your insightful comments and constructive suggestions. We appreciate the opportunity to clarify and enhance the statistical analysis section of our manuscript. Regarding your concern about the Cox proportional hazard model and the lack of detailed explanation for model selection and hypothesis testing, we would like to provide the following additional information: (1) model selection and hypothesis testing: The Cox proportional hazards model was chosen for our multivariate analysis due to its ability to handle censored survival data, which is common in studies involving long-term follow-up. This model allows us to estimate the hazard ratio (HR) for each variable while accounting for the time-to-event nature of the data. In our study, we first performed univariate Cox regression analysis to identify potential prognostic factors associated with OS and CSS. Variables that exhibited a P-value of less than 0.05 in the univariate analysis were considered candidates for inclusion in the multivariate model. This step was crucial for reducing the dimensionality of the model and focusing on the most relevant predictors. Subsequently, we constructed a multivariate Cox proportional hazards model to assess the independent effects of these variables on OS and CSS. The model was adjusted for potential confounders, including demographic and clinical characteristics, to ensure that the estimated HRs were accurate and unbiased. Hypothesis testing within the Cox model was conducted using the Wald test, which tests the null hypothesis that the coefficient (and thus the HR) for a given variable is equal to zero. (2) handling missing data: Missing data is a common challenge in large databases such as the SEER database. To address this issue, we adopted a hybrid approach that integrates multiple imputation (MI) and the random forest technique. MI was used to generate plausible values for missing data points based on the observed data. This method allowed us to create multiple imputed datasets, which were then analyzed separately using the Cox proportional hazards model. The results from these analyses were combined using standard rules for multiple imputation to produce final estimates that account for the uncertainty introduced by the imputation process. In addition, the random forest technique was employed to assess the importance of variables and to identify potential interactions that might affect the imputation process. This approach helped us to ensure that the imputed values were reasonable and consistent with the underlying data distribution. (3) criteria for choosing variables: Variables were selected for inclusion in the Cox model based on clinical relevance and statistical significance in the univariate analysis. We considered demographic factors (e.g., age, sex, race), tumor characteristics (e.g., grade, size, AJCC stage, T and N stages), and treatment-related factors (e.g., radiation, chemotherapy) as potential predictors of survival outcomes. To minimize selection bias, we used PSM to create comparable groups of patients who received LR and RR. This methodology allowed us to control for confounding variables and to isolate the effect of surgical approach on survival outcomes.

**[Revised manuscript]**

**[Statistical analysis]**

Statistical analysis was performed using R software (version 4.1.0). Categorical variables were presented as counts and percentages, and chi-square tests were used to assess differences among groups. For continuous data that deviated from a normal distribution, the median and interquartile range (IQR) were reported, and the Mann-Whitney U test was applied for comparisons. To ensure comparability between the LR and RR groups, a 1:1 PSM approach was implemented. Variables included in the PSM model were sex, age, race, tumor grade, marital status, tumor size, AJCC stage, T and N stages, radiation status, and chemotherapy status. A caliper width of 0.01 was used to match patients. Survival analyses were conducted using the Kaplan-Meier method, and survival curves were compared with the log-rank test. To evaluate the impact of various factors on OS and CSS, Cox proportional hazards models were employed for both univariate and multivariate analyses. The choice of the Cox proportional hazards model was based on its ability to handle censored data, which is common in survival analysis, and its capability to estimate hazard ratios (HRs) for the effect of predictors on the hazard of the event (i.e., death). The proportional hazards assumption was tested using Schoenfeld residuals, and no violations were found. For the univariate analysis, each variable was individually assessed for its association with OS and CSS. Variables with a P-value less than 0.05 in the univariate analysis were considered candidates for inclusion in the multivariate model. The multivariate Cox proportional hazards model was then constructed using a stepwise backward elimination approach, retaining variables that were statistically significant at the 0.05 level. To address missing data, which is a common challenge in large databases such as the SEER database, a hybrid approach combining multiple imputation (MI) and the random forest technique was adopted. Multiple imputation was used to generate plausible values for missing data points, and the random forest algorithm was employed to impute missing values based on the observed data and the relationships among variables. This approach aimed to minimize bias and improve the accuracy of the statistical analyses. Hazard ratios (HRs) and their corresponding 95% confidence intervals (CIs) were reported for all variables included in the multivariate Cox models. Statistical significance was determined by a P-value threshold of less than 0.05.

**Comment 2**

Supplement the related content of postoperative complications: increase the analysis of the incidence of postoperative complications and explore its relationship with the long-term survival results of patients. You can set a section in the results section, or present this section separately, so that the overall analysis of the patients after treatment can be more complete.

**Response 2**

Thank you for the reviewer's valuable feedback. We appreciate the suggestion to supplement the related content regarding postoperative complications and to analyze their incidence and relationship with long-term survival outcomes of patients. In response, we would like to clarify that our study is based on the SEER database. Unfortunately, the SEER database does not provide detailed information on postoperative complications. This limitation has indeed been acknowledged in the discussion section of our paper, where we have highlighted the need for future studies to incorporate such data. Despite this limitation, we understand the importance of postoperative complications in the overall prognosis of patients with PNEC. We agree that analyzing the incidence of postoperative complications and their impact on long-term survival would provide a more comprehensive understanding of the outcomes after different surgical approaches. However, given the constraints of the current dataset, we are unable to perform this analysis directly. We do hope that future studies, possibly utilizing prospectively collected data or other databases that include postoperative complication details, will be able to address this gap in knowledge.

**Comment 3**

Although stratified analysis was conducted, there may be a small sample size in some subgroups (such as age, sex, tumor size, etc.), which may affect the statistical significance of the results. It is suggested that the author provide more detailed information about subgroup analysis and discuss its clinical importance in the results section.

**Response 3**

Thank you for your insightful comments and valuable suggestions on our manuscript. We appreciate your recognition of our efforts in conducting a stratified analysis to compare the long-term survival outcomes between patients receiving LR and RR for PNEC without distant metastasis. Regarding your concern about the potential small sample size in some subgroups, we acknowledge that this is indeed a limitation of our study. As you pointed out, subgroups such as age, sex, and tumor size may have had insufficient sample sizes to detect statistically significant differences. However, we would like to emphasize that our primary objective was to compare the OS and CSS between the LR and RR groups after PSM, and our main findings remain robust, showing no significant differences in long-term OS (*P* = 0.746) or CSS (*P* = 0.634) between the two groups. To address your suggestion for more detailed information on subgroup analysis, we have now included additional details in the results section. Specifically, we have provided the number of patients in each subgroup and the corresponding P-values for OS and CSS comparisons. Although some subgroups had relatively small sample sizes, the consistency of the results across all subgroups, including those with larger sample sizes, supports our conclusion that LR and RR exhibit similar prognoses in patients with PNEC without distant metastasis. Furthermore, we have discussed the clinical importance of our subgroup analysis in the results section. We have highlighted that even in subgroups where the sample size was small, the lack of significant differences in OS and CSS between the LR and RR groups suggests that the choice of surgical approach may not significantly impact long-term survival outcomes in these patients. This finding is clinically relevant as it provides evidence to support the use of LR as a potentially adequate treatment option for patients with PNEC, especially in cases where RR may carry higher risks or be technically challenging.

**[Revised manuscript]**

**[Subgroup analysis]**

After PSM, both the LR group and the RR group exhibited comparable OS and CSS (*P* > 0.05). To further explore the consistency of these findings across different patient characteristics, we conducted subgroup analyses based on sex, age, race, grade, marital status, tumor size, AJCC stage, T stage, N stage, radiation, and chemotherapy. In these subgroup analyses, we observed that the OS and CSS remained comparable between the LR and RR groups across all stratification factors (*P* > 0.05). It is important to note that while some subgroups had relatively smaller sample sizes (e.g., certain age groups, specific tumor sizes), the statistical non-significance consistently observed across all subgroups suggests that the primary finding of similar prognoses between LR and RR is robust. The clinical importance of these findings lies in their implication for treatment decision-making. Our study suggests that for patients with PNEC without distant metastasis, the choice between LR and RR may not significantly impact long-term survival outcomes. This is particularly relevant in clinical scenarios where preserving organ function and minimizing surgical morbidity are crucial considerations. Figure 3 and Figure 4 provide detailed information on the comparisons among different subgroups, illustrating the consistency of our findings across various patient characteristics. These figures visually represent the lack of significant differences in OS and CSS between the LR and RR groups across all subgroups analyzed.

Reviewer #2: The manuscript is updated but not fully readable, and a better definition of study population and clinical protocols is needed especially defining patient characteristics and early clinical outcomes. The authors should revise their report and include the following aspects:

\# Major issues

**Comment 1**

How did the authors decide to analyze all together PNEC? As they certainly known, there is a reason to have not considereded the increasing risk for making a pancreatico-anastomosis following pancreatico-duodenecetomy (PD) into the clinical practice and why?

**Response 1**

Thank you for your insightful question regarding our decision to analyze all patients with PNEC together and our consideration of the risks associated with pancreatico-anastomosis following pancreaticoduodenectomy (PD) in clinical practice. In our study, we decided to analyze all patients with PNEC without distant metastasis together because our primary objective was to compare the long-term survival outcomes between LR and RR, regardless of the specific surgical technique used within the RR group (which may include PD in some cases). Our focus was on the OS and CSS differences between the two surgical approaches, rather than delving into the intricacies of each individual surgical procedure or its associated complications. Regarding the increasing risk of pancreatico-anastomosis following PD, we acknowledge that this is an important consideration in clinical practice. However, our study was designed to assess the long-term survival outcomes of patients based on the surgical approach (LR vs. RR), rather than to evaluate the specific surgical techniques or their complications. The decision to include patients who underwent PD as part of the RR group was based on the fact that PD is one of the radical surgical options for treating PNEC, and our aim was to compare the overall survival outcomes between radical and local resection approaches. It is worth noting that our study did not delve into the details of postoperative complications or the specific techniques used during surgery, as these were beyond the scope of our research question. However, we recognize the importance of considering such factors in clinical decision-making and patient management. In conclusion, our study was designed to compare the long-term survival outcomes of patients with PNEC without distant metastasis who underwent LR versus RR. While we acknowledge the risks associated with pancreatico-anastomosis following PD, our focus was on the overall survival differences between the two surgical approaches, rather than on the specific surgical techniques or their complications. We hope this clarifies our rationale for analyzing all patients with PNEC together in our study.

**Comment 2**

How did this direct their clinical management during the post-operative course? The order of events and the decision-making regarding this are not fully clear from the report.

**Response 2**

Thank you for raising the question regarding the clinical management of patients during the postoperative course and the decision-making process in our study. We understand that the report may not have fully clarified these aspects, and we would like to provide further clarification. In our study, our primary objective was to compare the long-term survival outcomes between patients with PNEC who received LR and those who underwent RR. The focus of our analysis was on the surgical approach and its impact on survival, rather than on the specific clinical management during the postoperative course. However, it is important to note that the postoperative management of patients with PNEC typically involves a multidisciplinary approach, including close monitoring for complications, pain management, nutritional support, and follow-up care. The specific management strategies may vary depending on the patient's overall condition, the extent of the surgery, and any postoperative complications that may arise. Regarding the decision-making process in our study, the choice of surgical approach (LR or RR) was based on the clinical judgment of the treating physicians and the specific characteristics of each patient's tumor. Our study did not interfere with or dictate the clinical management of the patients. Instead, we retrospectively analyzed the data to compare the long-term survival outcomes between the two surgical approaches. In clinical practice, the decision to perform LR or RR would take into account multiple factors, including the tumor size, location, grade, and stage, as well as the patient's overall health status and preferences. Our study provides evidence that in patients with PNEC without distant metastasis, LR and RR exhibit similar prognoses, which may inform clinical decision-making and suggest that LR may be an adequate treatment option for these patients. We hope this clarifies our approach to clinical management during the postoperative course and the decision-making process in our study. If you have any further questions or concerns, please do not hesitate to let us know.

**Comment 3**

Description of surgical techniques in terms of surgical experience, and of commonly used pre- and post-operative pancreatic function has to be included in a specific sub-heading.

**Response 3**

We greatly appreciate your insightful suggestion regarding the inclusion of a specific sub-heading to describe surgical techniques in terms of surgical experience and commonly used pre- and post-operative pancreatic function. Your comment highlights an important aspect of surgical studies that should be adequately addressed to provide a comprehensive understanding of the surgical approaches. However, we would like to point out that our study is based on data retrieved from the SEER database. While the SEER database provides valuable information on patient demographics, tumor characteristics, and survival outcomes, it does not include detailed descriptions of surgical techniques, surgical experience, or pre- and post-operative pancreatic function management. Given the nature of our data source, we acknowledge that we may not have access to the specific details that you have suggested. Nonetheless, we understand the importance of this information and will make every effort to discuss the limitations of our study in this regard. We will also emphasize that our study focuses on the comparison of long-term survival outcomes between LR and RR for PNEC without distant metastasis, using the best available data from the SEER database. We hope that our findings will contribute to the existing knowledge on this topic and provide valuable insights for future research. Thank you again for your constructive feedback. We will carefully consider your suggestions and make the necessary revisions to improve the clarity and completeness of our manuscript.

**[Revised manuscript]**

**[Discussion]**

Our research encounters several constraints. Initially, our analysis is retrospective and depends on the SEER database, which inherently bears the risk of inconsistent and biased data. Secondly, the SEER database is deficient in thorough information pertaining to postoperative complications, specific chemotherapy protocols, disease recurrence, and the individualized criteria for surgical method selection. These factors could significantly affect patients' long-term outcomes. Thirdly, this study lacks detailed information on surgical techniques, surgical experience, and pre- and post-operative pancreatic function management, which may provide a comprehensive understanding of the surgical approaches. Lastly, it's essential to recognize that the SEER database mainly mirrors the American healthcare system, and medical practices can differ widely among various regions and cultures. Hence, prudence is required when generalizing our study's results to other geographic or cultural settings. However, to our understanding, this study represents the most extensive comparison of long-term outcomes between LR and RR treatments for PNEC conducted thus far. Additionally, we utilized PSM to mitigate potential biases and performed subgroup analyses to assess the impact of different factors on patient prognosis.

**Comment 4**

Did the authors recognize study translational relevance and specific significant impact for the Health System?

**Response 4**

Yes, the authors do recognize the translational relevance and specific significant impact of this study for the health system. Our research compares the long-term survival outcomes between LR and RR for PNEC without distant metastasis, aiming to provide evidence-based guidance for clinical decision-making. The findings of our study suggest that LR and RR exhibit similar prognoses in this patient population. This has important implications for the health system, as it indicates that LR, which may be less invasive and carry fewer risks and costs, could be an adequate treatment option for certain patients with PNEC. This could potentially lead to more personalized treatment plans, reduced healthcare expenditures, and improved patient outcomes. Furthermore, our study highlights the independent prognostic risk factors for overall survival and cancer-specific survival, which can aid clinicians in identifying high-risk patients and tailoring treatment strategies accordingly. This contributes to a more efficient and effective use of healthcare resources, ultimately benefiting the health system as a whole. In summary, our study not only advances the understanding of surgical approaches for PNEC but also has practical implications for clinical practice and healthcare policy, emphasizing the importance of considering LR as a viable treatment option for selected patients.

\# Minor observations

**Comment 5**

If present, what were differences in post-operative surgical site infections?

**Response 5**

The study did not specifically investigate differences in post-operative surgical site infections (SSIs) between patients who received LR and RR for PNEC. The primary focus of our research was to compare long-term survival outcomes, including OS and CSS, between the two surgical approaches. However, it is worth noting that surgical site infections are an important aspect of post-operative care and can significantly impact patient recovery and outcomes. In general, radical resections may involve a larger incision and more extensive tissue manipulation, which could potentially increase the risk of SSIs compared to local resections. Nonetheless, the incidence and severity of SSIs can be influenced by various factors, including patient characteristics, surgical technique, perioperative management, and institutional practices. To address the reviewer's concern, we acknowledge that post-operative SSIs are an important consideration in the surgical management of PNEC patients. Future studies could specifically investigate the incidence and risk factors for SSIs in this patient population, as well as the impact of SSIs on long-term survival outcomes. Such research would provide valuable insights into optimizing surgical care and improving patient outcomes for PNEC.

**Comment 6**

It is mandatory to update the discussion with recent clinical experiences of specific interventions and with a better description of possible stopping rules or discontinuation criteria to be arranged by the future RCT investigators.

**Response 6**

We greatly appreciate the reviewer's insightful suggestion to update the discussion with recent clinical experiences of specific interventions and to provide a better description of possible stopping rules or discontinuation criteria for future RCT investigators. We fully recognize the importance of keeping our discussion section current and relevant to the evolving landscape of PNEC treatment. In response to the first part of the comment, we would like to highlight that our paper already incorporates recent clinical experiences related to the surgical management of PNEC. Specifically, we have referenced a range of studies (citations 8-18) that have contributed to our understanding of the efficacy and outcomes of different surgical approaches, including LR and RR. These references provide a comprehensive overview of the current state of knowledge in this field and support our findings. Regarding the second part of the comment, we understand the importance of outlining possible stopping rules or discontinuation criteria for future RCT investigators. However, we believe that a detailed discussion of these criteria may be beyond the scope of our current study, which primarily focuses on comparing long-term survival outcomes between LR and RR in patients with PNEC without distant metastasis. At this stage, we have chosen not to delve deeply into the specifics of stopping rules or discontinuation criteria for several reasons. Firstly, the design and implementation of such criteria are highly context-specific and depend on a variety of factors, including the study objectives, patient population, and available resources. Secondly, the development of these criteria typically involves extensive consultation with stakeholders, including clinicians, statisticians, and ethical committees, and may require a separate study or protocol to be fully elaborated. That said, we acknowledge the reviewer's concern and recognize the value of providing guidance for future RCT investigators. Therefore, we plan to address this topic in future work, possibly through a separate publication or as part of a larger research initiative. In the meantime, we encourage future investigators to consider the relevant ethical and methodological considerations when designing their studies and to consult with experts in the field to establish appropriate stopping rules and discontinuation criteria. In conclusion, we appreciate the reviewer's suggestions and have taken them into account in our response. We believe that our paper already incorporates recent clinical experiences related to PNEC treatment, and we plan to further explore the topic of stopping rules and discontinuation criteria in future work. Thank you again for your valuable feedback.

Journal requirements:

When submitting your revision, we need you to address these additional requirements.

1. Please ensure that your manuscript meets PLOS ONE's style requirements, including those for file naming. The PLOS ONE style templates can be found at

https://journals.plos.org/plosone/s/file?id=wjVg/PLOSOne_formatting_sample_main_body.pdf and

https://journals.plos.org/plosone/s/file?id=ba62/PLOSOne_formatting_sample_title_authors_affiliations.pdf

We ensure that our manuscript meets PLOS ONE's style requirements.

2. Thank you for stating the following financial disclosure:

“This work was supported by the Science and Technology Development Program of Suzhou (SKYXD2022041, SYWD2024322 and SYWD2024077).”

Please state what role the funders took in the study. If the funders had no role, please state: "The funders had no role in study design, data collection and analysis, decision to publish, or preparation of the manuscript."

If this statement is not correct you must amend it as needed.

Please include this amended Role of Funder statement in your cover letter; we will change the online submission form on your behalf.

We amended the statement and included this amended Role of Funder statement in our cover letter.

3. Please include captions for your Supporting Information files at the end of your manuscript, and update any in-text citations to match accordingly. Please see our Supporting Information guidelines for more information: http://journals.plos.org/plosone/s/supporting-information.

We include the caption for our Supporting Information file at the end of our manuscript.

Yours sincerely,

Qiao Z
2025-02-01
